# Association of Chronic Spontaneous Urticaria With Anxiety and Depression in Adolescents: A Mediation Analysis

**DOI:** 10.3389/fpsyt.2021.655802

**Published:** 2021-09-07

**Authors:** Yuzhou Huang, Yi Xiao, Danrong Jing, Jie Li, Jianglin Zhang, Xiang Chen, Minxue Shen

**Affiliations:** ^1^Department of Dermatology, Xiangya Hospital, Central South University, Changsha, China; ^2^National Clinical Research Center for Geriatric Disorders (Xiangya Hospital), Changsha, China; ^3^Hunan Engineering Research Center of Skin Health and Disease, Changsha, China; ^4^Hunan Key Laboratory of Skin Cancer and Psoriasis, Changsha, China; ^5^Department of Detmatology, Shenzhen People's Hospital, The Second Clinical Medical College, Jinan University, The First Affiliated Hospital, Southern University of Science and Technology, Shenzhen, China; ^6^Department of Social Medicine and Health Management, Xiangya School of Public Health, Central South University, Changsha, China

**Keywords:** chronic spontaneous urticaria, itching, sleep disturbance, mediation effect, depression, anxiety

## Abstract

**Background:** Chronic spontaneous urticaria (CSU) is related to psychiatric comorbidities. It is not clear whether the relationship is affected by modifiable factors.

**Objectives:** To investigate whether the effect of CSU on anxiety and depression in adolescents is mediated by the symptoms of itching and sleep disturbance.

**Methods:** Questionnaire survey was conducted among newly enrolled college students. Dermatologists diagnose skin diseases, including CSU, during health examination. Anxiety and depression were measured by the Generalized Anxiety Disorder Scale and Patient Health Questionnaire, respectively. Sleep quality was measured by the Pittsburgh Sleep Quality Index. The symptoms of itching were measured by the numeric rating scale. According to the hypothesis, the mediating effect model was put forward and the structural equation model is used to build the mediation effect model. The mediation effect model was proposed according to the hypothesis and established using a structural equation model.

**Results:** A total of 2,358 students with no history of systemic disease and no pruritus disease (except CSU) were included in the analysis. A total of 393 CSU patients were included, and 1,965 healthy controls were selected based on age and sex matching. CSU was significantly associated with both anxiety and depression when the symptoms of itching and sleep quality were not modeled. A mediation model was proposed as CSU → itching → sleep disturbance → anxiety or depression. Itching and sleep quality mediated 65.4 and 77.6% of CSU's effects on anxiety and depression, respectively, and CSU had no significant direct effect on anxiety or depression in the mediation models.

**Conclusions:** The associations of CSU with anxiety and depression were mediated by the symptoms of itching and sleep disturbance. Effectively reducing the symptoms of itching thereby could increase natural sleep, which can further treat the emotional disorders among patients with CSU.

## Introduction

Chronic urticaria (CU) is a common skin disease characterized by the occurrence of wheals (hives), angioedema, or both lasting more than 6 weeks (1). CU can be classified into chronic spontaneous urticaria (CSU) and inducible urticaria (IU) (1–3). CSU occurs spontaneously with no obvious cause, while IU occurs when the formation of hives is reproducible after specific stimulus (4). CU affects 0.5–1% of the general population (5) and 0.1–0.3% of children (6). However, the prevalence of CU in adolescents is underappreciated. Globally, urticaria contributes to 4.7 million age-standardized disability-adjusted life years and 4.7 years lived with disability (7). Many studies have shown that patients with CSU often experience mental complications (8–10). The most common mental disorders observed in CSU patients are depression, anxiety, and somatoform disorders (8). The wide range of estimates of psychosocial factors among patients with CU was 16–96% (11). A recent meta-analysis found that CSU patients are six times more likely to suffer from anxiety and depression than healthy people (12). So, the psychiatric disorders associated with CSU should be taken seriously.

CSU is characterized by transient itching. Itching symptoms in patients with CSU have been described as stinging, tickling, and burning. Because of unpredictable attacks of pruritus and swelling, urticaria can seriously affect their quality of life (13). Itching is one of the main symptoms and the most important cause of sleep disturbance in patients with CSU. The severity of itching in CSU patients is associated with severe impairment of sleep quality (14–16). CSU severely affects sleep and quality of life, leading to difficulties related to work, family activities, social life, family relationships, sex, hobbies, and holidays.

Previous research has found that itching and sleep disturbance have a significant effect on mental disorders. Most clinical studies have revealed a correlation between mental disorders and chronic pruritus in patients with skin disorders. Chronic itching is associated with a high incidence of stress, anxiety, depression, and even suicidal thoughts, leading to major defects in quality of life (17–21). Sleep disturbance is significantly associated with increased risk of depression (22).

It is unknown whether CSU causes emotional problems through the symptoms of itching and sleep disturbance. Our study aims to investigate the mediation effect of the symptoms of itching and sleep disturbance on emotional problems in adolescents with CSU.

## Methods

### Study Design

The College Student Skin Health Survey (CSSHS) (23) is a 4-year dynamic cohort of college students aiming to investigate skin health, diseases, and risk factors in adolescents. In the current study, we proposed a hypothesized pathway from CSU to emotional problems and tested the hypothesis using the baseline data of the cohort. Students from all over the country were admitted to four universities in four provinces (Hunan, Hubei, Fujian, and Xinjiang) and immediately performed health examinations and questionnaire surveys. All newly enrolled students who agreed to participate were classified as a cluster in September 2017 and September 2018. Participants that reported severe underlying diseases or the use of NSAIDs (24), or were diagnosed as other pruritic skin diseases, were excluded from the statistical analysis. We continuously selected patients with CSU from baseline data and matched healthy controls at a ratio of 1:5 based on age and sex matching.

### Questionnaire and Measurements

During the physical examination, the University Student Affairs Department conducted a web-based questionnaire survey within 1 day. The questionnaire was self-reported (in computer rooms) and the questionnaire took 15 min to fulfill on average. The questionnaire was comprised of demographic information, history of diseases that might be associated with skin health, history of allergy, cigarette smoking, alcohol drinking, intake of soft drinks, water intake, food taste preference, defecation, sport, sleep quality, anxiety, depression, bath habit, skincare, and sun exposure. Anxiety and depression were measured by the two-item Generalized Anxiety Disorder Scale (GAD-2) and two-item Patient Health Questionnaire (PHQ-2), respectively. Sleep quality was measured by the Pittsburgh Sleep Quality Index (PSQI). The intensity of itching was measured by the numeric rating scale (NRS) of itching. For GAD-2, PHQ-2, PSQI, and NRS of the symptoms of itching, a higher score signified a greater level of anxiety, depression, sleep disturbance, and the symptoms of itching, respectively.

### Clinical Evaluation and Diagnosis

Diagnosis of skin diseases and inquiry of disease history were performed by certified dermatologists during the health examination. Clinical manifestation, disease history, and family history of participants were inquired, and physical examinations were conducted to further diagnose CSU. During the health examination, qualified dermatologists conducted a skin disease diagnosis and medical history inquiry. The doctors asked the participants clinical manifestations, medical history, and family history and performed physical examinations to further diagnose CSU. CSU was diagnosed as wheals (hives), angioedema onset for 6 weeks or more with no obvious cause.

### Statistical Analysis

Continuous data were presented as mean ± standard deviation, and between-group difference was tested using analysis of variance (ANOVA). Multiple comparisons were performed with the Least Significant Difference (LSD) *t*-test. Categorical data was presented as number (%), and the between-group difference was tested using the chi-square test.

**Figure 1:**
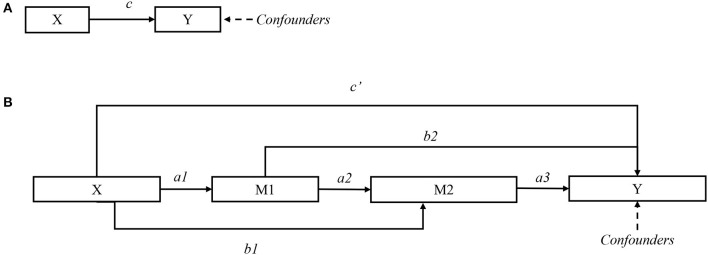
Directed acyclic graph for mediation effect model. **(A)** Total effect *c*. **(B)** Direct effect *c'*; and mediation effects *a1* × *b2, b1* × *a3*, and *a1* × *a2* × *a3*.

The mediation effect model was performed to investigate whether CU affect emotional problems through itch and sleep disturbance. We proposed a model with a three-path mediated effect as: CSU (X) → itching (M1) → sleep disturbance (M2) → anxiety or depression (Y). As shown in Figure 1A, the total effect of predictor X on outcome Y is *c*; in Figure 1B, the direct effect of X on Y is *c'*, and the mediation effect of M1 and M2 is calculated as *a1* × *b2* + *b1* × *a3* + *a1* × *a2* × *a3*. The significance of the mediation effect was tested using the Bootstrap method. If the direct effect is insignificant and the mediation effect is significant, then mediators have a complete mediation effect on the outcome. *P* < 0.05 was considered statistically significant for all tests. Statistical analysis was performed in SPSS software 23.0 (IBM, NY, USA).

## Results

Based on screening of baseline data, we identified a total of 393 CSU patients, and a healthy control group of 1,965 was selected based on age and gender matching. The characteristics of the students stratified by CSU are shown in Table 1. Household income was associated with CSU.

**Table 1:** Characteristics of participants, stratified by chronic spontaneous urticaria.

		**Chronic spontaneous**		
		**urticaria**		
**Characteristics**	**Total**	**Case**	**Control**	***P***
Geographic region^a^				
North	246 (10.4)	38 (9.7)	208 (10.6)	0.198
Northeast	127 (5.4)	21 (5.3)	106 (5.4)	
East	573 (24.3)	74 (18.8)	499 (25.4)	
Central	664 (28.2)	121 (30.8)	543 (27.6)	
South	217 (9.2)	45 (11.5)	172 (8.8)	
Southwest	302 (12.8)	67 (17.0)	235 (12.0)	
Northwest	229 (9.7)	27 (6.9)	202 (10.3)	
Age (years)		18.3 ± 0.7	18.3 ± 0.7	1.0
Gender				
Male	1,128 (47.8)	188 (47.8)	940 (47.8)	1.0
Female	1,230 (52.2)	205 (52.2)	1,025 (52.2)	
Ethnicity				
Han	2,095 (89.0)	347 (89.0)	1,748 (89.0)	0.704
Other	263 (11.0)	46 (11.0)	217 (11.0)	
Annual household income (yuan)				
< 10,000	185 (7.8)	18 (4.6)	167 (8.5)	0.001
10,000–29,999	470 (19.9)	63 (16.0)	407 (20.7)	
30,000–49,999	390 (16.5)	69 (17.6)	321 (16.3)	
50,000–99,999	571 (24.2)	100 (25.4)	471 (24.0)	
100,000–199,999	529 (22.4)	101 (25.7)	428 (21.8)	
≥200,000	213 (9.0)	42 (10.7)	171 (8.7)	

a *North: Beijing, Tianjin, Hebei, Shanxi, Inner Mongolia; Northeast: Liaoning, Jilin, Heilongjiang; East: Shanghai, Jiangsu, Zhejiang, Anhui, Fujian, Jiangxi, Shandong, Taiwan; Central: Henan, Hubei, Hunan; South: Guangdong, Guangxi, Hainan, Hong Kong, Macao; Southwest: Chongqing, Sichuan, Guizhou, Yunnan, Tibet; Northwest: Shaanxi, Gansu, Qinghai, Ningxia, Xinjiang*.

In crude estimation, as shown in Table 2, students with CSU had significantly higher levels of the symptoms of itching, sleep disturbance, anxiety, and depression. Results remained consistent when dichotomizing the outcomes by certain cutoffs.

**Table 2:** Comparison of itch, sleep quality, anxiety, and depression among adolescents with chronic spontaneous urticaria and those without.

	**CSU**		**Effect size**		
**Outcomes**	**Yes**	**No**	**Difference (95% CI)**	**OR (95% CI)**	***P***
Itch NRS	2.07 ± 2.13	1.02 ± 1.35	1.06 (0.90–1.22)		<0.001
Itch NRS ≥3	129 (32.8)	239 (12.2)		3.53 (2.75–4.53)	<0.001
PSQI	4.20 ± 3.14	3.54 ± 2.90	0.67 (0.35–0.99)		<0.001
PSQI ≥6	120 (30.5)	404 (20.6)		1.70 (1.31–2.16)	<0.001
GAD-2	0.99 ± 1.27	0.78 ± 1.10	0.21 (0.09–0.33)		0.001
GAD-2 ≥3	34 (8.7)	99 (5.0)		1.79 (1.19–2.68)	0.005
PHQ-2	0.97 ± 1.27	0.79 ± 1.14	0.18 (0.06–0.31)		0.005
PHQ-2 ≥3	34 (8.7)	109 (5.5)		1.61 (1.08–2.41)	0.020

*CSU, chronic spontaneous urticaria; OR, odds ratio; CI, confidence interval; NRS, numeric rating scale; GAD, Generalized Anxiety Disorder; PHQ, Patient Health Questionnaire; PSQI, Pittsburgh sleep quality index; NRS, numeric rating scale*.

**Figure 2:**
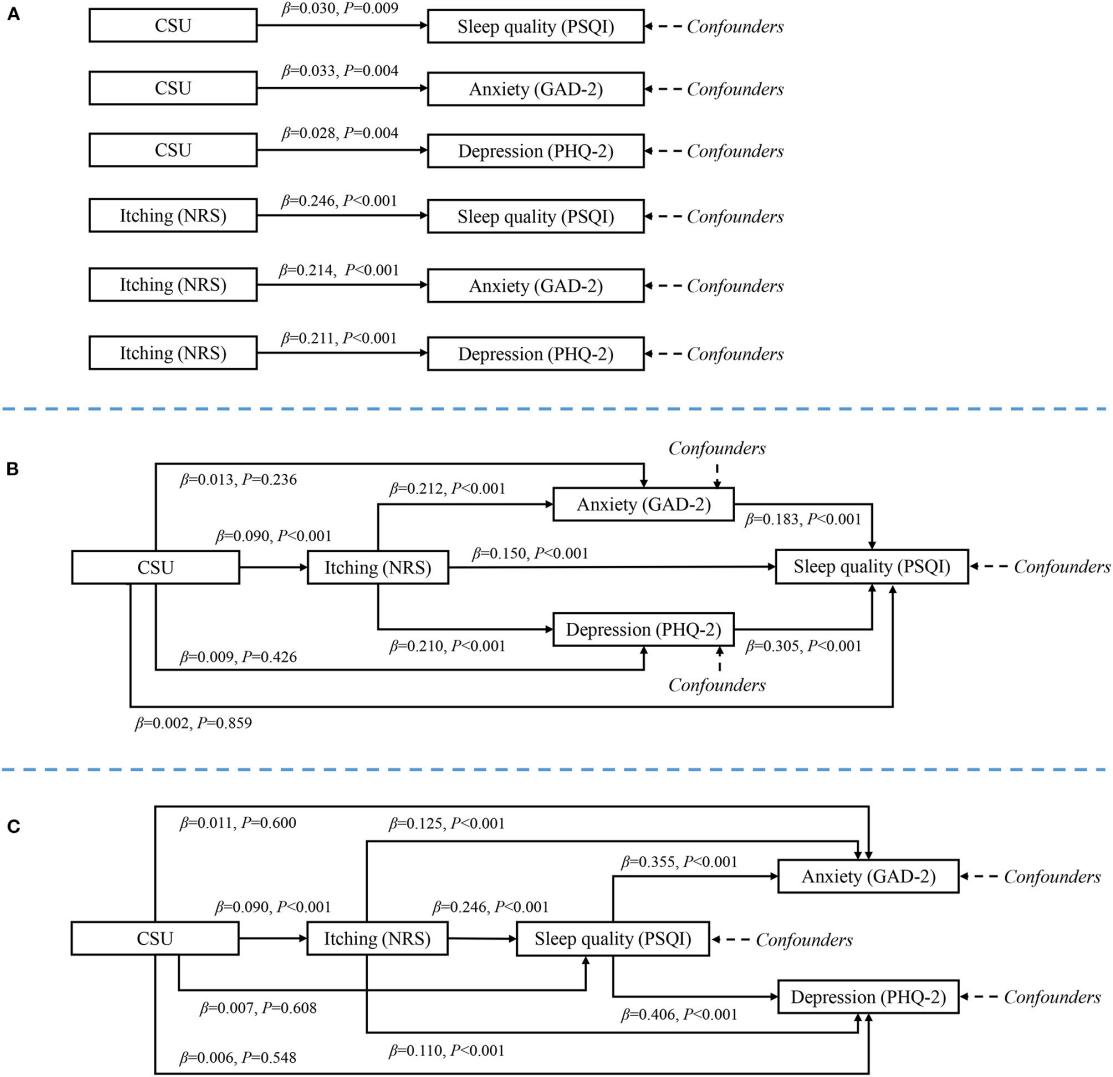
Mediation model from chronic spontaneous urticaria to emotional problems. **(A)** Total effects, adjusted for confounders but not adjusted for mediators. **(B)** Direct and mediation effects of one-mediator model. **(C)** Direct and mediation effects of two-mediator model. Chronic spontaneous urticaria had significant total effect on sleep quality, anxiety and depression; but when mediators were modeled, urticaria had no significant direct effect on sleep quality, anxiety and depression. Itching and sleep quality had significant mediation effect.

As shown in Figure 2A, CSU had significant total effects on sleep quality and emotional problems after adjustment for geographic region, age, gender, household income, food allergy, and drug allergy. One-mediator model is shown in Figure 2B: the direct effect of CSU on anxiety and depression was not significant; the symptoms of itching mediated 87.1 and 110.0% of CSU's effect on anxiety and depression, respectively (both *P* < 0.001). The two-mediator model is shown in Figure 2C: the direct effect of CSU on sleep quality, anxiety, and depression was not significant; the symptoms of itching and sleep disturbance mediated 95.7 and 116.7% of CSU's effect on anxiety and depression, respectively (both *P* < 0.001). The total effect, direct effect, and mediation effect are summarized in Table 3.

**Table 3:** Effect of predictors and mediators for sleep quality, anxiety, and depression.

			**Total effect^a^**		**Direct effect^b^**		**Mediation effect^c^**		
**Predictor (X)**	**Mediator (M)**	**Outcome (Y)**	**Size^d^**	***P***	**Size^d^**	***P***	**Size (95%CI)^d^**	***P***	**%**
Itching	Sleep quality	Anxiety	0.238	<0.001	0.123	<0.001	0.113 (0.098, 0.128)	<0.001	47.5
Itching	Sleep quality	Depression	0.252	<0.001	0.143	<0.001	0.110 (0.095, 0.125)	<0.001	43.7
Itching	Anxiety	Sleep quality	0.271	<0.001	0.168	<0.001	0.098 (0.055, 0.141)	<0.001	36.2
Itching	Depression	Sleep quality	0.271	<0.001	0.164	<0.001	0.103 (0.058, 0.148)	<0.001	38.0
CSU	Itching	Sleep quality	0.084	<0.001	0.015	0.458	0.069 (−0.067, 0.205)	<0.001	82.1
CSU	Itching	Anxiety	0.070	0.001	0.010	0.644	0.061 (0.005, 0.117)	<0.001	87.1
CSU	Itching	Depression	0.060	0.004	−0.005	0.818	0.066 (0.005, 0.0.127)	<0.001	110.0
CSU	Itching → sleep quality	Anxiety	0.070	0.001	0.003	0.870	0.067 (−0.008, 0.142)	<0.001	95.7
CSU	Itching → sleep quality	Depression	0.060	0.004	−0.011	0.558	0.070 (−0.007, 0.147)	<0.001	116.7
CSU	Itching → anxiety	Sleep quality	0.084	<0.001	0.011	0.546	0.073 (−0.116, 0.262)	<0.001	86.9
CSU	Itching → depression	Sleep quality	0.084	<0.001	0.017	0.359	0.068 (−0.115, 0.251)	<0.001	81.0

*CSU, chronic spontaneous urticaria*.

a *Total effect refers to the effect of predictor (X) on outcome (Y), adjusted from confounders but not mediators (M)*.

b *Direct effect refers to the effect of predictor (X) on outcome (Y), adjusted for confounders and mediators (M)*.

c *Mediation effect refers to the product of standardized coefficients of predictor to mediator and mediator to outcome, and the significance is tested by Bootstrap method. Percentage of mediation effect is calculated as: mediation effect/total effect ×100%*.

d *Expressed as standardized regression coefficient*.

## Discussion

We proposed a mediation model from CSU to the symptoms of itching, sleep disturbance, and finally emotional problems in adolescents. The effects of CSU on anxiety and depression were fully mediated by the symptoms of itching and sleep disturbance, and CSU had no significant direct effect on anxiety or depression.

It is reported that at least 30% of patients with skin diseases have mental and psychosocial disorders (25–29). A systematic review found that the prevalence of depression in psoriasis patients ranged from 9 to 62%, and the prevalence of anxiety ranged from 11 to 43% (30). A recent study showed that depression, anxiety, and suicidal ideation are more common among patients with atopic dermatitis (31). The impact of CSU on quality of life was similar to that of cardiovascular disease (32). A systematic review showed that psychosocial factors had a prevalence of 46% in CSU patients (11). Our research is consistent with previous research results. The risk of anxiety and depression in urticaria patients is 1.79 times and 1.61 times that of normal people, respectively.

It is mentioned in “the European S2k Guideline on Chronic Pruritus” that all patients with urticaria have itching (33). It has also been reported that CSU is the source of itching in about 3% of children (34). A recent multicenter study found that compared with patients without itching, the depression and suicidal ideation of patients with skin diseases were strongly correlated with the presence of itching (35). This shows that the mental health problems of patients with skin diseases are largely related to itching. Other studies have confirmed this (36). One presumed reason for this correlation is that itching is associated with skin inflammation, which induces serotonin networks in the brain, leading to depression and anxiety (37, 38). Therefore, the symptoms of itching can be used as a major cause of mediating the occurrence of anxiety and depression in patients with CSU.

CSU has been reported to be associated with sleep disorders. Previous studies showed that patients with longer duration of CSU reported more sleep disturbance, tiredness, and irritability (39). Caine found that comparison of the Nottingham Health Profile scores shows that sleep disruption was a bigger problem for patients with CU. O'Donnell et al. reported that 38% of CU patients reported marked sleep disruption, and another 54% had sleep interference (40). A study in Singapore showed that many patients had difficulty falling asleep and often woke in the night (14). The CU-Q2oL scores from the German population showed that the greatest burdens of QoL were sleep, itching/embarrassment, and mental status (41).

Longitudinal studies have shown that subjective sleep disturbance is the main risk factor for the first-onset and recurrent depressive episodes of young and old people in the future (22, 42–44). Poor sleep quality can produce negative cognitions and emotions that are not conducive to sleep, such as anxiety and anger. As these vicious cycles continue, the risk of depression increases (45). One study showed that people with persistent insomnia have an average increased risk of depression by 3.7 times compared with those without insomnia (46). A meta-analysis found that people with insomnia have twice the risk of depression compared to people without sleep disorders (22). A systematic study of the relationship between adolescents' sleep duration and emotions found that sleep duration has a significant negative impact on the various emotional states of healthy adolescents (47). In a recent meta-analysis, it was found that lack of sleep may bring the risk of anxiety-related symptoms (48). Classic H1-antihistamines increase daytime sleepiness and reduce sleep quality scores, which have a negative impact on mood. The second-generation antihistamines have little effect on mood and are better than H1-antihistamines (49). Therefore, we believe that increasing sleep quality and natural sleep are more beneficial for anxiety and depression in patients with urticaria.

According to previous studies, the relationship between CSU and sleep disorders and the relationship between sleep disorders and anxiety and depression have been found. We can speculate that sleep disorder is a mediator of anxiety and depression in CSU patients.

In our study, when modeling itching as the only mediator, it mediated 87.1 and 110.0% of CSU's effect on anxiety and depression, respectively. By contrast, when modeling itching as the first and sleep quality as the second mediator, they mediated 95.7 and 116.7% of CSU's effect on anxiety and depression, respectively. The two-mediator model explained significantly greater variations than the one-mediator model. This is a further evidence of the importance of sleep disorders in CSU-associated emotional problems.

The study has limitations. First, the study was a case-control study, and the level of evidence limits the capability of causal relationship inference. Longitudinal observations are needed to confirm this proposed mediation model. Second, the study population was limited to highly homogeneous adolescents aged around 18 and having a similar educational background. The generalizability of the findings might be limited to other populations. Third, owing to the feasibility of study implementation, anxiety and depression were only measured by two brief tools that were generally applied in clinical settings. Fourth, the urticaria activity score (UAS) scale and treatment status were not included in the CSU patients' inquiries, which may have a certain influence on the results.

The study also has strengths. First, this was a population-based epidemiologic survey among adolescents, and it had a higher level of evidence than the hospital-based studies. Second, the mediation model from CSU to the symptoms of itching, sleep disturbance, and emotional problems was clinically rational; the findings might provide potentially effective methods of an intervention targeting on the mediators. Third, the questionnaire survey and diagnosis for CSU were conducted using standardized methodologies.

In summary, we proposed a mediation effect model to test whether CSU was associated to anxiety and depression through mediators including the symptoms of itching and sleep disturbance. We combined the known evidences from literatures and validated the hypothesis in adolescents. Our research suggests that effectively reducing the symptoms of itching thereby could increase natural sleep, which can reduce emotional problems among CSU patients.

## Data Availability Statement

The raw data supporting the conclusions of this article will be made available by the authors, without undue reservation.

## Ethics Statement

This study was conducted according to the guidelines laid down in the Declaration of Helsinki. All procedures involving patients were approved by the institutional research ethics boards of Xiangya Hospital, Central South University (Changsha, China). Informed consent was obtained from all students before the investigation.

## Author Contributions

YH, YX, and DJ performed the dermatological examinations. Senior dermatologists JZ and JL were responsible for quality control for diagnoses. MS and YH analyzed the data and drafted the manuscript. MS and YX designed the questionnaire. MS, JL, JZ, and XC designed the study and critically reviewed and revised the manuscript. MS and XC obtained the funding. All authors participated in the field survey and data collection and gave final approval to the version submitted for publication.

## Funding

This work was supported by the Ministry of Science and Technology of People's Republic of China (grant# 2016YFC0900802), Central South University (grant# 202045005), and the Program of Introducing Talents of Discipline to Universities (111 Project, No. B20017).

## Conflict of Interest

The authors declare that the research was conducted in the absence of any commercial or financial relationships that could be construed as a potential conflict of interest.

## Publisher's Note

All claims expressed in this article are solely those of the authors and do not necessarily represent those of their affiliated organizations, or those of the publisher, the editors and the reviewers. Any product that may be evaluated in this article, or claim that may be made by its manufacturer, is not guaranteed or endorsed by the publisher.
